# Labor Analgesia in a Patient With Beals Syndrome: A Case Report of Management Challenges

**DOI:** 10.7759/cureus.79302

**Published:** 2025-02-19

**Authors:** João Laranjeira, Vasyl Katerenchuk, Joana Duarte, Muriel Lérias-Cambeiro, Filipa Lança

**Affiliations:** 1 Anesthesiology, Unidade Local de Saúde de Santa Maria, Lisboa, PRT; 2 Anesthesiology, Unidade Local de Saúde da Arrábida, Setúbal, PRT

**Keywords:** anesthesiology, beals syndrome, combined spinal/epidural, congenital contractural arachnodactyly, labor analgesia, neuraxial analgesia, neuraxial labor analgesia, neuraxial technique, obstetric anesthesia, regional analgesia

## Abstract

Beals syndrome, also known as Beals-Hetch syndrome or congenital contractural arachnodactyly, is an autosomal dominantly inherited, rare connective tissue disorder characterized by flexion contractures, arachnodactyly, camptodactyly, severe kyphoscoliosis, and, less frequently, cardiovascular features. We describe the successful management of labor analgesia in a pregnant woman with Beals syndrome. During labor, a combined spinal/epidural technique was performed uneventfully, and intermittent top-ups were administered as needed at lower-than-usual volumes. Eutocic delivery occurred without complications, and both mother and baby were discharged three days later. Given the sparse literature about obstetric patients with Beals syndrome, we summarize the main anesthetic challenges and suggested approaches.

## Introduction

Beals syndrome, also known as Beals-Hetch syndrome or congenital contractural arachnodactyly, is an autosomal dominantly inherited, rare, connective tissue disorder first described by Beals and Hecht in 1971 [1]. It is caused by a mutation in the fibrillin-2 gene (FBN2) on chromosome 5q23, which causes a change in the structure of the fibrillin-2, a protein that contributes to providing support in elastic and non-elastic connective tissue [2,3]. Flexion contractures, arachnodactyly, camptodactyly, severe kyphoscoliosis, abnormal pinnae, and muscular hypoplasia characterize the disease. It can also present with cardiovascular involvement, including aortic root dilation or mitral valve prolapse, and ophthalmologic abnormalities, including heterotopia [3,4].

Beals syndrome shares phenotypic features with Marfan syndrome, although ocular and cardiovascular features are less frequent in Beals syndrome. The molecular basis for this clinical overlap is well established with both diseases resulting from mutations in two homologous genes (FBN2 vs. FBN1) [2-4]. Given this overlap in phenotype, the incidence of Beals syndrome is unknown, and its prevalence is difficult to estimate. Parental somatic and germline mosaicism have been observed [3].

Management of patients with Beals syndrome is symptomatic. Camptodactyly and contractures involving other joints may spontaneously improve with time, but residual camptodactyly remains. Cardiac and ophthalmologic evaluations are recommended, as well as routine physical examination for spinal deformity. Early intervention for scoliosis can prevent morbidity later in life. There is no evidence of a shortened lifespan [3-4].

The syndrome's associated facial changes can lead to difficult airway management, while cardiac and pulmonary involvement may further complicate anesthetic intervention. Additionally, challenging neuraxial blockade techniques and difficult positioning can pose significant obstacles [3,5]. To the best of our knowledge, there is a scarcity of published literature addressing the anesthetic management of pregnant women with Beals syndrome. In this context, we report the successful anesthetic approach in a pregnant patient with this syndrome, highlighting the specific challenges encountered and the strategies employed to ensure a positive maternal and fetal outcome.

The case described below was previously presented as a poster at Euroanaesthesia 2024 on May 26, 2024.

## Case presentation

**Figure 1:**
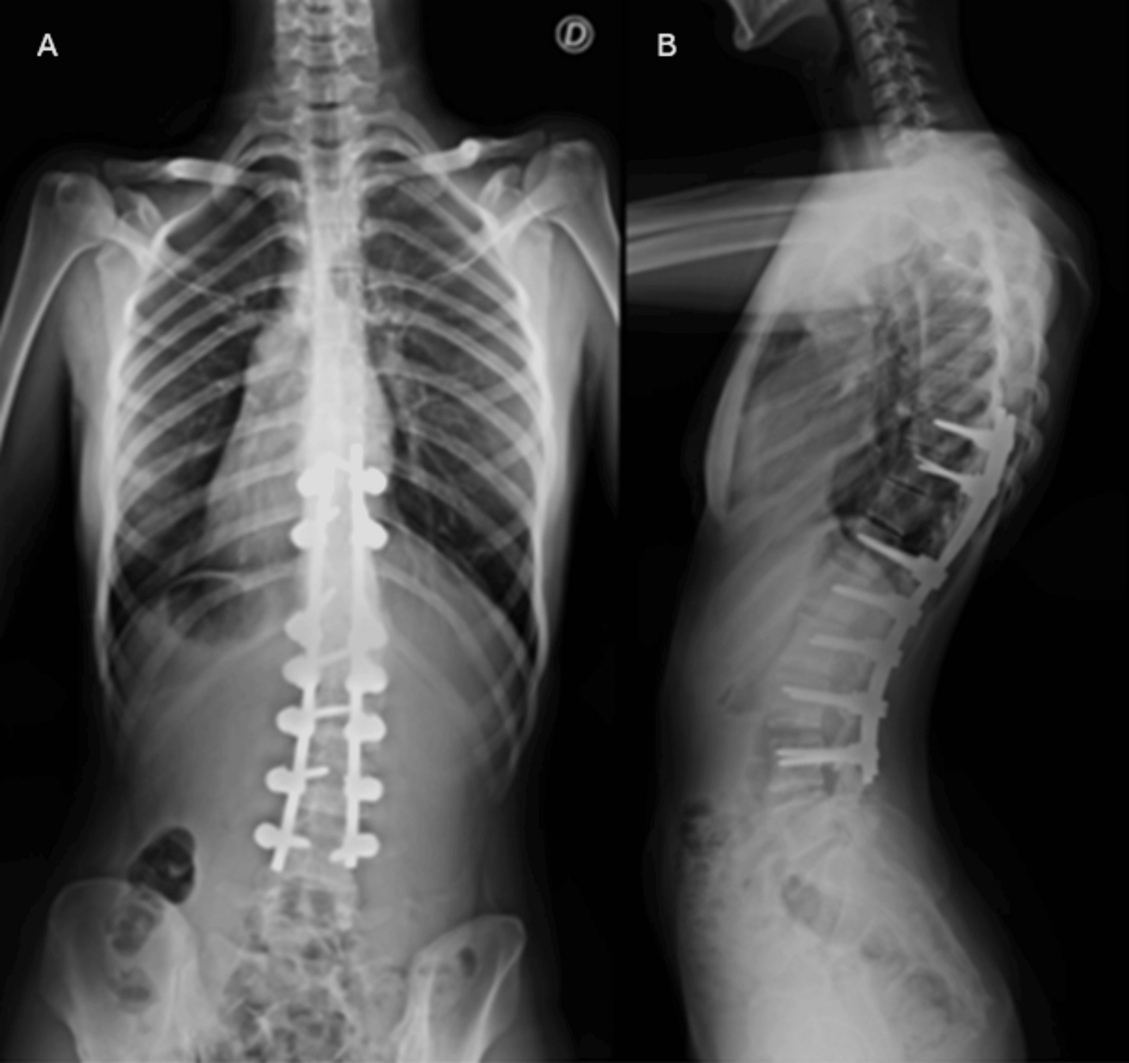
(A) Axial and (B) lateral X-ray of the patient's spine Both figures highlight surgical material following posterior spinal surgery from T9 to L4. In the figures, we can see that several vertebral bodies have fixation screws and that two longitudinal rods extend from T9 to L4. The presence of this surgical material may hinder the search for the epidural space.

We present the case of a 21-year-old primigravida diagnosed with Beals syndrome at three months of age. She was born with facial dysmorphia, an ogival palate, crumpled ears, camptodactyly of the third, fourth, and fifth hand fingers, and bilateral clubfoot. Her sister had also been diagnosed with Beals syndrome. During her childhood and adolescence, she developed severe scoliosis, muscular hypoplasia, and congenital muscle contractures. She denied any cardiac features, although she did not remember the date of her last echocardiographic evaluation. She had previously undergone bilateral astragalus corrective surgery and posterior spinal surgery from D9 to L4 under general anesthesia, both without perioperative complications. The spinal surgery yielded favorable functional and radiological outcomes. Figure 1 shows the patient has two longitudinal fixation rods, one on each side, stabilizing the vertebrae from T9 to L4. The presence of this fixation hardware may complicate the identification of an appropriate puncture site by altering normal anatomy and reducing available space. Additionally, it may hinder optimal patient positioning by limiting interspinous space opening, thereby making epidural space localization more challenging.

The patient was referred to our tertiary hospital for genetic counseling, obstetric follow-up, and anesthetic evaluation. Anesthesia consultation was performed at 32 weeks of gestation. On physical examination, she had a marfanoid habitus with long limbs, sternal asymmetry, and scoliosis. The airway assessment revealed a Mallampati score of I, an inter-incisor distance superior to 3 cm, a thyromental distance superior to 6 cm, and normal neck mobility. The patient had good functional capacity (5.3 METs, using the Duke Activity Status Index, although this value could be underestimated due to limitations associated with pregnancy), with no symptoms associated with exertion or rest and a peripheral saturation of 98%. The other vital signs were within normal parameters. In addition, the neurological examination and coagulation study were normal. The transthoracic echocardiogram (routinely performed one year before pregnancy) was unremarkable. The patient was classified as an American Society of Anesthesiologists Physical Status II. We explained to the patient that there were no contraindications to performing neuraxial analgesia/anesthesia. Still, there remained a possibility that the technique could fail and that she may have a higher risk of complications (such as accidental puncture of the dura mater). Alternatives to neuraxial techniques, namely intravenous analgesia (and its risks and potential benefits), were also explained. After consideration, the patient agreed that neuraxial analgesia should be attempted.

At 39 weeks of gestation, she was admitted to labor and requested analgesia. A central neuraxial block was chosen, and as per our department’s standard practice, a combined spinal-epidural technique was performed at the L3/L4 interspace, estimated using anatomical landmarks using the needle-through-needle approach. The epidural space was located through a loss of resistance to saline technique at 5 cm, and 5 cm of the catheter was left in the epidural space. No difficulties were encountered with needle or catheter insertion. Sufentanil 5 micrograms was administered in the subarachnoid space. Later, the epidural catheter was tested with 4 ml of ropivacaine 0.2%. Hemodynamic parameters were stable. There was no motor or sensory blockade. Taking into account that the patient had an instrumented spine, no additional dose of local anesthetic was administered, and pain was assessed 20-30 minutes later regarding pain relief. Subsequent ropivacaine 0.2% intermittent top-ups were administered as needed at lower than usual volumes (only 5 ml required for adequate pain relief). The commonly used dose in our clinical practice is approximately 10 mL of 0.2% ropivacaine (20 mg), effectively achieving the desired pain relief in most pregnant women. The duration of labor was approximately 11 hours, and the neuraxial technique was done soon after admission. Pain control was achieved, and the patient was comfortable during that period, although scores were not numerically quantified. The anesthesiologist performed regular assessments; top-up doses were necessary every two hours. Eutocic delivery occurred of a male newborn, Apgar 9-10-10. The catheter was removed uneventfully soon after labor. Both mother and baby were discharged three days later.

## Discussion

Since Beals syndrome is extremely rare and its exact incidence is unknown, there is limited information in the literature about the anesthetic management of these patients. When such patients require surgery, scoliosis correction in children is the most commonly reported procedure [5-8]. To our knowledge, the literature on obstetric anesthesia in patients with Beals syndrome is lacking. Considering this, we summarize the main anesthetic challenges and suggest approaches to managing these patients.

Beals syndrome may present in a phenotypic multitude of ways, so we highly recommend an anesthesia consultation before admission. Given the risk of complex airway features, a careful airway assessment should be performed, with reported cases of difficult airways in children with Beals syndrome [5-8]. However, the patient in this case did not exhibit signs of a difficult airway. Therefore, in cases where a difficult airway is anticipated (though not necessarily in the patient described in this case), a regional anesthetic approach should be prioritized. However, thorough preparation for airway management is essential, including the option of awake fiberoptic endotracheal intubation in high-risk patients [9]. If an emergent C-section was required, a videolaryngoscope and a tube with a stylet were prepared and readily available. Additionally, restrictive lung disease may also be present as a consequence of severe scoliosis, and functional capacity should always be assessed [10]. While this patient, post-scoliosis correction, presented with good functional capacity and no symptoms of restrictive lung disease, a high index of suspicion would prompt referral to pulmonology for evaluation, including pulmonary function testing. Although less often than in patients with Marfan syndrome, patients with Beals syndrome may present cardiac features. Aortic root dilation, mitral valve prolapse, and septal defects have been reported [5,10]. We recommend a preoperative electrocardiogram and a recent echocardiogram to exclude cardiac features. Furthermore, imaging studies were reviewed in this patient with prior spinal instrumentation (Figure 1).

Regarding anesthetic technique, there are no contraindications to using neuraxial analgesia or anesthesia. However, the possibility of failure and a potential increase in complications, such as dural puncture, should be communicated in advance to establish realistic expectations. The technique may be challenging due to severe scoliosis, spine deformities, flexion contractures in multiple joints, and spinal fusion with stiff rods that can impede pre-procedural patient positioning by limiting spinal flexion and access to interspinous spaces. Additionally, scarring of the epidural space due to previous back surgery can cause unpredictable local anesthetic spreading in the epidural space, resulting in patchy, unilateral, or different-than-expected levels of the block and even reduced analgesic efficacy [5,11]. For this reason, the lowest possible dose should be started in the presence of the anesthesiologist, with incremental doses as needed and frequent tailored pain assessments, as in this case. In case of technical difficulty, ultrasound (to identify lumbar anatomy and interspace level) and rescue spinal can be considered.

Beals syndrome, which shares some phenotypic similarities with Marfan syndrome, also shares some challenges with the latter. Namely, regarding neuraxial techniques that can be more difficult due to deformities of the lumbar spine or a history of lumbar instrumentation, as previously mentioned [12]. However, Marfan syndrome presents additional concerns, such as dural ectasia, which increases the risk of dural puncture during epidural catheter placement and may lead to ineffective spinal anesthesia [12]. A unique challenge with Marfan is related to the dilation of the aortic root, and pregnancy can increase the risk of aortic dissection [12].

## Conclusions

Beals syndrome patients can present a variety of anesthetic challenges given their facial abnormalities, cardiologic features, challenging neuraxial blockade technique, and difficult positioning. Ideally, these patients should be referred to a tertiary maternity unit to receive a multidisciplinary approach, including specialties such as anesthesiology, obstetrics, genetics, neurology, and pulmonology. Management of these patients requires a thorough airway and cardiac evaluation during anesthesia consultation, as well as individualized local anesthetic titration during labor analgesia. Despite its challenges, an early neuraxial technique may be crucial, reducing the need for emergent airway management, which can be particularly challenging in some patients with Beals syndrome in the event of an emergency cesarean section.
